# The assessment of thiol-disulfide homeostasis and ıschemia-modified albumin levels in patients with acromegaly

**DOI:** 10.1007/s11102-025-01519-y

**Published:** 2025-04-05

**Authors:** Emre Urhan, Canan Sehit Kara, Esra Fırat Oguz, Salim Neselioglu, Ozcan Erel, Hamiyet Donmez Altuntas, Fahri Bayram

**Affiliations:** 1 Department of Endocrinology, Erciyes University Medical School, Kayseri, Turkey; 2 Department of Medical Biochemistry, Ankara City Hospital, Ankara, Turkey; 3 Department of Medical Biochemistry, Yildirim Beyazit University Medical School, Ankara, Turkey; 4 Department of Medical Biology, Erciyes University Medical School, Kayseri, Turkey

**Keywords:** Acromegaly, Oxidative stress, Thiol-disulfide homeostasis, Thiol, Disulfide, Ischemia-modified albumin

## Abstract

**Purpose:**

Data regarding the relationship between acromegaly and oxidative stress (OS) remain limited. Dynamic thiol-disulfide homeostasis (TDH) is vital for antioxidant protection, and ischemia-modified albumin (IMA) serves as a marker of OS. This study aimed to measure serum TDH parameters and IMA levels in acromegaly patients, comparing them with healthy controls.

**Methods:**

This cross-sectional study consecutively included 81 patients and 55 controls, matched for age, gender, and body mass index. Serum levels of native thiol, total thiol, and disulfide (TDH parameters) were measured using the automated spectrophotometric method developed by Erel and Neselioglu, along with serum IMA levels.

**Results:**

In patients, serum native and total thiol levels were significantly lower (p = 0.005 and p = 0.007), while serum IMA levels were significantly higher (p = 0.001). Disulfide levels were similar. Patients with active disease (N = 32), patients in remission (N = 49), and controls (N = 55) were compared. In post-hoc analyses; serum TDH parameters and IMA levels were similar in remission and active disease patients. Native and total thiol levels were significantly lower in patients in remission compared to controls (p = 0.01 and p = 0.04). IMA levels were significantly higher in patients in remission compared to controls (p = 0.04). Serum thiol levels positively correlated with serum insulin-like growth factor-1 levels and negatively with age and disease duration, while age independently exerted a negative impact on serum thiol levels.

**Conclusion:**

Our findings may indicate increased OS in the acromegalic process, which may contribute to the development of acromegaly and its related complications and comorbidities.

## Introduction

Acromegaly is a rare endocrine disease characterized by elevated serum growth hormone (GH) and insulin-like growth factor-1 (IGF-1) levels, usually originating from a pituitary adenoma [1]. It is a multisystemic process accompanied by many increased mortalities and morbidities such as endothelial dysfunction, hyperglycemia, hypertension, hyperlipidemia, cardiovascular diseases, and malignancy [2].

Oxidative stress (OS) arises from an imbalance between oxidant and antioxidant mechanisms, involving decreased antioxidant protective mechanisms and/or increased free radicals and reactive oxygen species (ROS), causing damage to biological structures in the body [3]. OS plays a role in ROS mediated-cell damage in various processes such as cancer, inflammation, infection, atherosclerosis, and aging. Enzymatic or non-enzymatic antioxidants try to balance the damage caused by OS to the biological structures of the body [4].

Thiols are organic compounds formed by bonding sulfur and hydrogen atoms to a carbon atom and are a key component of antioxidant system. They are also known as mercaptans and serve as a type of radical scavenger [5, 6]. The plasma thiol pool primarily consists of albumin thiols and, to a lesser extent, low molecular weight thiols such as cysteine, homocysteine, glutathione, cysteinylglycine, and gamma-glutamylcysteine [5]. In the presence of OS, thiols attempt to minimize this toxic damage. If body thiols levels decrease, antioxidant capacity decreases and disulfide levels are expected to increase. In the early stages of OS, oxidant radicals oxidize the sulfur-containing amino acids of thiol groups, forming reversible disulfide bonds, and thus, thiol levels decrease. If OS is eliminated, disulfide bonds will be converted back into thiol groups. This way, thiol-disulfide homeostasis (TDH) is maintained [7, 8]. The dynamic thiol-disulfide balance plays a critical role in antioxidant protection, apoptosis, intracellular signal transmission, detoxification, and enzymatic regulatory activity [9]. There are direct and indirect methods to measure OS in the body. Determining thiol-disulfide levels indirectly indicates OS. In 2014, Erel and Neselioglu developed a novel automated spectrometric method to measure collectively thiol-disulfide levels with high sensitivity and specificity [10].

Ischemia-modified albumin (IMA) is a biochemical test used in conditions associated with acidosis, free radical damage, hypoxia, and ischemic processes. It is the oxidative modified form of albumin and is one of the markers of OS. In situations of hypooxygenation caused by free radicals, serum IMA levels increase [11, 12].

Although data on the relationship between acromegaly and OS is limited, various oxidative status abnormalities have been identified [13–17]. Endothelial dysfunction is a well-defined process in the course of acromegaly and is associated with increased ROS and/or decreased antioxidant capacity. OS may act as a trigger or contributor to the development of acromegaly or disease-related complications [17, 18]. Physiological levels of IGF-1 and GH increase endothelial nitric oxide synthase (NOS) expression, and consequently, NO production. In vivo and in vitro studies, it has been demonstrated that endothelial NOS is activated through the GH and IGF-1 pathway [19]. This process inhibits ROS production, aiming to limit OS [20, 21]. In active acromegaly, decreased levels of NO and increased OS markers have been reported [13, 22].

The utility of OS markers has been investigated in the etiopathogenesis, diagnosis, treatment, subgroup differentiation, and treatment monitoring of many diseases in clinical practice and studies [23]. However, there are limited studies on this aspect in acromegaly. In the present study, for the first time in the literature to the best of our knowledge, we aimed to measure serum TDH parameters and IMA levels in acromegaly patients and compare them with those in healthy controls, and to investigate their relationship with disease subgroups and related parameters.

## Materials and methods

### Study participants

This study was conducted with the approval of the Erciyes University Ethics Committee, and informed consent was obtained from the participants.

Patients diagnosed with acromegaly and healthy controls, matched with patients for age, gender, and body mass index (BMI), followed at the Erciyes University Faculty of Medicine Endocrinology Clinic between 2019 and 2022 were consecutively included in this cross-sectional study. The diagnosis of acromegaly was based on elevated serum IGF-1 levels according to age and gender, and the lack of GH suppression during a 75 g oral glucose tolerance test (OGTT) (defined as the lowest GH level during the test being higher than 0.4 ng/mL) [24]. Active acromegaly was defined as elevated serum IGF-1 levels according to age and gender, and the lack of GH suppression during the OGTT-GH test in untreated patients. Acromegaly in remission was defined as normal serum IGF-1 levels according to age and gender, a random serum GH level below 1 ng/mL, and GH suppression during the OGTT-GH test in untreated patients [25].

The exclusion criteria for the study were determined as being under 18 years of age, having a history of malignancy, hematological and inflammatory diseases, presence of active infection, pregnancy and breastfeeding, liver and renal failure, history of neurodegenerative diseases, active smoking and alcohol use, history of cardiovascular events, presence of untreated hormonal deficiencies, and use of antioxidant drugs and vitamins.

Patients' demographic information, duration of acromegaly diagnosis, serum IGF-1 and other pituitary hormone levels, treatments given for acromegaly including history of pituitary surgery, medical treatment and radiotherapy, glycemic status (fasting blood glucose (FBG) ≥ 126 mg/dL or haemoglobin A1c (HbA1c) ≥ 6.5% were considered as diabetic, FBG < 100 mg/dL and HbA1c < 5.7% were considered non-diabetic, and intermediate values were considered prediabetic [26]), presence of hypertension and hyperlipidemia (hypercholesterolemia: low-density lipoproteins (LDL) level > 130 mg/dL and hypertriglyceridemia: triglyceride level > 150 mg/dL [27]), and treatments used for these conditions, liver and kidney function tests were recorded.

### Analytical measurements

Serum GH (ng/mL), IGF-1 (ng/mL, all ages and gender specific), prolactin (PRL) (Female: 2.8–29.2 ng/mL, male: 2.1–17.7 ng/mL), thyroid-stimulating hormone (TSH) (0.27–4.20 μU/mL), free triiodothyronine (FT3) (2–4.4 pg/mL), and free thyroxine (FT4) levels (0.93–1.97 ng/dL) were measured using the electrochemiluminescence immunoassay (ECLIA) technique with commercially available assay kits (Cobas; Roche Diagnostics, Germany) between 08.00 and 09.00 h. IGF-1 levels were adjusted based on the upper limit of normality (ULN) for age and gender.

### Serum TDH parameters and IMA measurements

Venous blood samples were collected from the participants in the early morning following overnight fasting, centrifuged at 1500 rpm for 10 min, and stored at -80 degrees Celsius until the evaluation process. The samples were analyzed simultaneously by the same person and device. The serum levels of native thiol, total thiol, and disulfide, which are parameters of TDH, were measured using the fully automated spectrophotometric method developed by Erel and Neselioglu, as previously described [10], and presented in µmol/L. Serum IMA levels were measured using the method developed by Bar-Or et al., and presented in IU/mL, as previously described [28].

### Statistical analysis

Statistical analyses were performed using IBM SPSS version 23.0 (IBM Inc, USA). Data were recorded as mean ± standard deviation (SD) or median (quartile 25–75%) depending on the distribution. The Shapiro–Wilk test was used to determine the distribution of the data. For comparisons between groups of quantitative variables, the independent samples t-test was used if the data were normally distributed. If the data were not normally distributed, the Mann–Whitney U test was used. Chi-square tests were performed for qualitative variables. One-way ANOVA was used to compare active acromegaly, acromegaly in remission, and healthy control groups. In post hoc analyses, the Games-Howell test was used when variances were not equal, and the Tukey HSD test was used when variances were equal. Pearson or Spearman analysis was used according to the data distribution for the correlation analysis. Multiple linear regression analysis was used to determine the effect of independent variables on serum thiol-disulfide parameters in acromegaly patients. A p-value of < 0.05 was considered statistically significant.

## Results

**Fig. 1:**
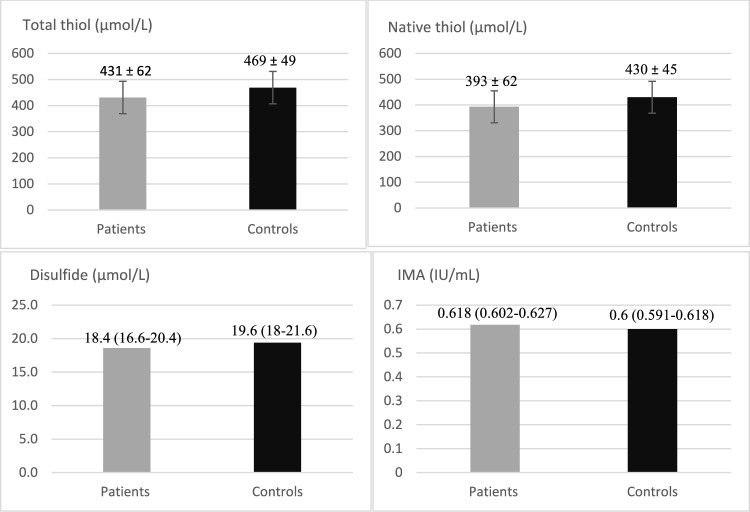
Graph of the comparison of parameters in acromegaly patients and healthy controls. Data are presented as mean ± standard deviation or median (quartiles 25–75%). Native thiol (µmol/L): p = 0.005, Total thiol (µmol/L): p = 0.007, Disulfide (µmol/L): p = 0.06, IMA (IU/mL): p = 0.001

The present study included 81 patients and 55 controls, and the groups were similar in terms of age (49.3 ± 10.5 vs 47.7 ± 11.8 years, respectively), gender (41 male, 40 female vs 29 male, 26 female, respectively), and BMI (30.9 ± 4.9 vs 29.1 ± 4.1 kg/m^2^, respectively). In acromegaly patients, serum HbA1c, FBG, and IGF-1/x ULN were significantly higher (p = 0.01, p = 0.02, and p = 0.001, respectively). In terms of serum TDH parameters, native thiol and total thiol levels were significantly lower in acromegaly patients compared to controls (393 ± 62 vs 430 ± 45 µmol/L, p = 0.005, and 431 ± 62 vs 469 ± 49 µmol/L, p = 0.007, respectively), while serum IMA levels were significantly higher (0.618 (0.602–0.627) vs 0.6 (0.591–0.618) IU/mL, p = 0.001). Disulfide levels were similar between the two groups (18.4 (16.6–20.4) vs 19.6 (18–21.6) µmol/L, p = 0.06). The demographic and biochemical results of the patients and controls are shown in Table 1. The graph comparing the parameters between acromegaly patients and healthy controls is shown in Fig. 1.

**Table 1:** Comparison of demographic and biochemical results of acromegalic patients and controls

Variables	Patients Group (N = 81)	Control Group (N = 55)	P Value
Age (years)	49.3 ± 10.5 Range (21–65)	47.7 ± 11.8 Range (19–65)	0.41
Gender	41 male, 40 female	29 male, 26 female	0.63
BMI (kg/m^2^)	30.9 ± 4.9	29.1 ± 4.1	0.57
HbA1c (%)	5.8 (5.4–6.5)	5.5 (5.3–5.7)	0.01
FBG (mg/dL)	100 (89–124)	94 (79–103)	0.02
LDL (mg/dL)	111 (95–135)	108 (92–137)	0.61
HDL (mg/dL)	50 (42–59)	47 (30–57)	0.06
Triglycerid (mg/dL)	124 (90–180)	118 (84–179)	0.12
Total cholesterol (mg/dL)	191 (173–221)	179 (153–212)	0.23
Albumin (mg/dL)	4.3 ± 0.1	4.3 ± 0.2	0.78
TSH (µU/mL)	1.3 (0.7–2.1)	1.1 (0.8–1.8)	0.35
FT4 (ng/dL)	1.3 (1.1–1.4)	1.4 (1.1–1.6)	0.19
Free T3 (pg/mL)	2.9 ± 0.3	3 ± 0.3	0.09
IGF-1 (x ULN)	0.89 (0.65–1.78)	0.43 (0.25–0.72)	0.001
Prolactin (ng/mL)	8.6 (6.5–10.3)	9.5 (6.8–11.8)	0.09
Native thiol (µmol/L)	393 ± 62	430 ± 45	0.005
Total thiol (µmol/L)	431 ± 62	469 ± 49	0.007
Disulfide (µmol/L)	18.4 (16.6–20.4)	19.6 (18–21.6)	0.06
IMA (IU/mL)	0.618 (0.602–0.627)	0.6 (0.591–0.618)	0.001

*BMI* Body mass index, *HbA1c* Haemoglobin A1c, *FBG* Fasting blood glucose, *LDL* Low-density lipoproteins, *HDL* High-density lipoprotein, *TSH* Thyroid-stimulating hormone, *FT4* Free thyroxine, *FT3* Free triiodothyronine, *IGF-1* Insulin-like growth factor-1, *ULN* Upper limit of normality, *IMA* Ischemia-modified albumin

Seventy-eight patients (96%) had a history of transsphenoidal surgery, and four (5%) of them also received radiotherapy, while three patients (4%) had not undergone transsphenoidal surgery. While 32 (39.5%) had active disease, 49 (60.5%) were in remission. In terms of glycemic status, 18 (22%) were diabetic, and 26 (32%) were prediabetic. Twenty-two patients (27%) had hyperlipidemia, and twenty-seven (33%) had hypertriglyceridemia. Twenty patients (25%) were hypertensive. The median disease duration was 5 (2–9.2) years (range: 0–20 years). Regarding medical treatment for acromegaly, 40 patients (49%) were not receiving treatment, while 41 (51%) were receiving treatment (27 with somatostatin analogs (SAs), 4 with cabergoline, 3 with a combination of SAs and cabergoline, 3 with a combination of SAs and pegvisomant, and 4 with a combination of SAs, cabergoline, and pegvisomant).

Patients with active disease (N = 32), patients in remission (N = 49), and controls (N = 55) were compared. Significant differences were found between the groups in terms of FBG, IGF-1/x ULN, native thiol, total thiol, and IMA levels (p = 0.04, p = 0.08, p = 0.01, p = 0.01, and p = 0.002, respectively). The disease duration was significantly longer in patients in remission compared to those with active disease (p = 0.001). In post-hoc analyses: FBG levels were significantly higher in patients in remission compared to controls (p = 0.02). LDL levels were significantly higher in patients in remission compared to those with active disease (p = 0.03). Serum TDH parameters and IMA levels were similar in patients in remission compared to those with active disease. IGF-1/x ULN were significantly higher in patients with active disease compared to those in remission and controls (p = 0.001). Serum prolactin levels and thyroid function tests were similar between the groups. Native and total thiol levels were significantly lower in patients in remission compared to controls (p = 0.01 and p = 0.04, respectively). IMA levels were significantly higher in patients in remission compared to controls (p = 0.04). Comparison of parameters according to acromegaly disease activity is shown in Table 2.

**Table 2:** Comparison of variables according to acromegaly disease activity

Variables	Active (A) Acromegaly (N = 32)	Remission (R) Acromegaly (N = 49)	Control (C) Groups (N = 55)
Age (years)	46.8 ± 13.1	51 ± 8.1	47.7 ± 11.8
Gender	18 male, 14 female	23 male, 26 female	29 male, 26 female
BMI (kg/m2)	31 ± 4.6	29.7 ± 3.9	29.1 ± 4.1
Disease duration (Years)	2.2 (0–5)	5.7 (3–10)	
HbA1c (%)	5.7 (5.4–6.3)	5.8 (5.5–6)	5.5 (5.3–5.7)
FBG (mg/dL)	98 (89–116)	104 (89–115)	94 (79–103)
LDL (mg/dL)	103 (86–125)	113 (99–150)	108 (92–137)
HDL (mg/dL)	47 (39–53)	52 (43–61)	47 (30–57)
Triglycerid(mg/dL)	133 (94–173)	121 (86–184)	118 (84–179)
Total cholesterol(mg/dL)	180 (148–204)	201 (178–232)	179 (153–212)
Albumin (mg/dL)	4.4 ± 0.1	4.3 ± 0.1	4.3 ± 0.2
IGF-1 (x ULN)	2.91 (1.6–3.12)	0.79 (0.57–0.86)	0.43 (0.25–0.72)
Native thiol (µmol/L)	402 ± 67	388 ± 58	430 ± 45
Total thiol (µmol/L)	441 ± 63	425 ± 62	469 ± 49
Disulfide (µmol/L)	18.3 (16–19.6)	18.5 (16.6–20.4)	19.6 (18–21.6)
IMA (IU/mL)	0.613 (0.6–0.629)	0.618 (0.6–0.628)	0.603 (0.591–0.615)

*BMI* Body mass index, *HbA1c* Haemoglobin A1c, *FBG* Fasting blood glucose, *LDL* Low-density lipoproteins, *HDL* High-density lipoprotein, *TSH* Thyroid-stimulating hormone, *FT4* Free thyroxine, *FT3* Free triiodothyronine, *IGF-1* Insulin-like growth factor-1, *ULN* Upper limit of normality, *IMA* Ischemia-modified albumin

Disease duration (Years): (A)vs(R) p = 0.001, FBG (mg/dL): (A)vs(R)vs(C) p = 0.04, (R)vs(C) p = 0.02, Total cholesterol(mg/dL): (A)vs(R) p = 0.008, IGF-1 (x ULN): (A)vs(R)vs(C) p = 0.008, (A)vs(R) p = 0.001, (A)vs(C) p = 0.001, Native thiol (µmol/L): (A)vs(R)vs(C) p = 0.01, (R)vs(C) p = 0.01, Total thiol (µmol/L): (A)vs(R)vs(C) p = 0.01, (R)vs(C) p = 0.04, IMA (IU/mL): (A)vs(R)vs(C) p = 0.002, (R)vs(C) p = 0.04

Patients with diabetes (N = 18), pre-diabetes (N = 26) and non-diabetes (N = 37), and controls (N = 55) were compared for serum TDH parameters and IMA levels. Significant differences were found between the groups in terms of native thiol and total thiol levels (p = 0.03 and p = 0.02, respectively). In post-hoc analyses: Native thiol levels were significantly lower in pre-diabetic (384 ± 61 µmol/L) and diabetic patients (388 ± 62 µmol/L) compared to controls (430 ± 45 µmol/L)(p = 0.005 and p = 0.02, respectively). Total thiol levels were significantly lower in pre-diabetic (418 ± 63 µmol/L) and diabetic patients (427 ± 59 µmol/L) compared to controls (470 ± 49 µmol/L). Native thiol and total thiol levels were similar in non-diabetic patients and controls. Native thiol and total thiol levels were similar among patients based on their glycemic status. Disulfide and IMA levels were similar among all groups.

Thiol-disulfide homeostasis parameters and IMA levels were compared by gender in both patients (41 male, 40 female) and controls (29 male, 26 female). No differences were found between males and females. TDH parameters and IMA levels were compared in patients receiving medical treatment (N = 41) and those not receiving treatment (N = 40), and no differences were found. There were no differences detected in the parameters based on the presence of hyperlipidemia, hypertriglyceridemia, and hypertension in patients.

Correlation analysis was performed. Native thiol levels were significantly positively correlated with total thiol levels (p = 0.001, r = 0.986) and IGF-1/x ULN (p = 0.001, r = 0.432), and significantly negatively correlated with age (p = 0.001, r = − 0.441) and disease duration (p = 0.01, r = − 0.271). Total thiol levels were significantly positively correlated with IGF-1/x ULN (p = 0.001, r = 0.403), and significantly negatively correlated with age (p = 0.001, r = − 0.454) and disease duration (p = 0.007, r = − 0.302). There were no significant correlations in the analysis of other parameters.

Multiple linear regression analyses in acromegalic patients showed that age was an independent negative predictor for serum native and total thiol levels (Beta = − 0.61, 95% CI: − 5.5 to − 1.6, p = 0.001, for both), and no significant effects were found with other variables.

## Discussion

In the present study, we evaluated serum TDH parameters using a simple and automated method and IMA levels, indicators of OS, in acromegalic patients and healthy controls. For the first time in the literature, we found that serum thiol levels were lower and IMA levels were higher in patients, which may provide evidence of increased OS in acromegaly patients.

Oxidative stress plays a fundamental and/or triggering role in the pathogenesis of many chronic diseases [4]. Studies on the relationship between acromegaly and OS are limited, and definitive conclusions have not yet been established. Physiological serum levels of IGF-1 and GH restrict OS. In acromegaly, increased IGF-1 levels lead to reduced endothelial NOS expression, resulting in decreased serum NO levels. Consequently, it is thought that increased ROS and/or decreased antioxidant mechanisms may lead to OS-related damage [17].

Ronconi et al. evaluated platelet NO levels and endothelial NOS expression as an antioxidant markers in active acromegalic patients and healthy controls, finding these levels to be lower in patients. However, the study had a small sample size, with only 13 patients. They suggested that this could increase the risk of atherosclerotic disease in acromegaly. Additionally, platelet NO levels were found to be negatively correlated with serum IGF-1 levels and disease duration [17]. In our study, we found that serum thiol levels were positively correlated with IGF-1/x ULN and negatively correlated with age and disease duration. Özısık et al. compared serum levels of glutathione peroxidase (GSH-Px), catalase, malondialdehyde (MDA), and superoxide dismutase (SOD) as indicators of OS in acromegalic patients in remission and healthy controls, finding similar levels between the two groups [14]. In contrast, we found that serum thiol levels were lower and IMA levels were higher in patients with remission compared to controls. İlhan et al. evaluated serum total antioxidant capacity (TAC), measured using the modified CUPRAC method, and SOD levels in acromegalic patients, including both active and remission patients, as well as in healthy controls. They found that these levels were lower in patients compared to controls. Compared to the controls, while TAC levels were lower in both active and remission patients, and serum SOD levels were similar with remission patients but lower in active patients. Both levels were similar among active and remission patients [15]. In our study, serum thiol and IMA levels were also similar between patients with active and remission disease.

Anagnostis et al. evaluated serum NO and glutathione levels and catalase activity as antioxidant markers, as well as serum thiobarbituric acid reactive substances (TBARS) levels as an oxidative marker, in predominantly active acromegalic patients and healthy controls. Antioxidant markers were lower in the patients, while TBARS levels were higher [29]. Tabur et al. measured serum prolidase activity, total oxidative status, and lipid hydroperoxide levels as an oxidative marker in acromegalic patients in remission and healthy controls. These levels were found to be higher in the patients [30]. Prolidase is a matrix metalloproteinase (MMP), and its activity is affected by OS, which increases MMP production [31]. They suggested that even when acromegaly goes into remission, OS-related damage may persist [30]. We also found lower serum thiol levels and higher IMA levels in patients in remission compared to healthy controls, suggesting that even though the disease is under control, the effects of OS may persist, leading to disease-related damage. Boero et al. compared some OS markers between active acromegalic patients and healthy controls. They found higher serum oxidized LDL levels and ceruloplasmin activity in the patients, while serum TBARS, SOD, paraoxonase 1, and myeloperoxidase levels were similar [13]. In another study, OS markers such as 8-hydroxy-2-deoxyguanosine (8-OHdG) and TBARS levels were evaluated in serum and in cardiomyocytes and vascular smooth muscle cells in the aorta of acromegalic rat models and healthy control rats. The levels were found to be higher in both the serum and tissues of the acromegalic rats. The researchers suggested that increased OS in acromegaly could potentially trigger atherosclerotic risks [22]. Bayram et al. evaluated cytokinesis-block micronucleus cytome assay parameters in peripheral lymphocytes as indicators of cytotoxicity and DNA damage, as well as serum 8-OHdG levels as a marker of oxidative DNA damage in active acromegalic patients and healthy controls. These parameters were found to be higher in the patients [16]. Yarman et al. assessed the levels of serum TBARS, a product of lipid peroxidation, in newly diagnosed acromegaly patients by evaluating their baseline levels at diagnosis and the levels obtained 4, 8, and 24 h after the subcutaneous administration of 100 µg of short-acting octreotide. Both the baseline and the post-octreotide levels were higher compared to healthy controls. Levels at 4 and 8 h were significantly lower than the baseline, while levels at 24 h showed a tendency to increase [32]. Nevertheless, TBARS levels may not serve as a specific marker of OS, as it can be influenced by substances other than the secondary products of lipid peroxidation [33].

Among TDH parameters, the balance between the oxidative side, disulfide, and the antioxidant side, thiol, which plays a fundamental role in reducing OS and preventing cellular damage, is essential for the physiological integrity of the body [5], and IMA is an indicator of OS [11]. In our study, we found that serum native and total thiol levels were lower and IMA levels were higher in acromegalic patients, while disulfide levels were similar. These results may suggest a decreased antioxidant system and increased ROS and OS in process of acromegaly disease. It remains unclear whether these changes are a consequence of acromegaly or if they are factors that trigger its development, making it difficult to make definitive interpretations. As a result, increased OS in acromegaly may play a role in endothelial dysfunction, atherosclerosis, elevated cardiovascular disease risk, increased malignancy, and the development of other comorbidities and complications. While an increase in the oxidant disulfide levels is expected, the similar levels observed may be a result of the effects of treatments used for primary disease management in acromegaly or from unforeseen heterogeneous differences between the patients and controls. In our study, a positive correlation was found between IGF-1/x ULN and thiol levels. Although it was expected that thiol levels would be lower in active patients, TDH parameters and IMA levels were similar in patients with active acromegaly and those in remission. Differences in disease duration, biochemical and demographic characteristics, types and durations of acromegaly treatments may affect these parameters and explain the similarity between groups. In remission-phase patients, serum thiol levels were lower, while serum IMA levels were higher compared to healthy controls. Thus, it may suggest that the OS process could continue even when acromegaly goes into remission. Serum thiol levels were negatively correlated with age and disease duration, with age having a significant negative impact on serum thiol levels. It is known that OS increases with age [34], and the decline in thiol levels with advancing age may also contribute to the OS process.

When acromegalic patients were evaluated according to glycemic status—classified as diabetic, prediabetic, and non-diabetic—the serum TDH parameters and IMA levels were similar across these groups. Compared to healthy controls, these levels were similar in non-diabetic patients, while serum thiol levels were lower in both prediabetic and diabetic patients. Glycemic status can affect the oxidant and antioxidant systems, as hyperglycemia may lead to an increase in the oxidant system and a decrease in the antioxidant system [35]. This process could result in the reduction of serum thiol levels, or the decrease in thiol levels could potentially trigger this process. Serum TDH parameters have been examined in a few studies involving diabetic patients. Decreased serum native thiol levels have been observed in both type 1 [36] and type 2 diabetes [35], while prediabetic patients have shown reductions in both serum native and total thiol levels [37]. Ates et al. found that thiol levels were negatively correlated with HbA1c levels in type 1 diabetic patients [38], and with FBG and HbA1c levels in prediabetic patients [37]. In our study, no correlation was found between serum TDH parameters, IMA levels, and glycemic parameters. The lower serum thiol levels observed in our acromegalic patients may be influenced by the presence of increased OS in the disease process, as well as the increased hyperglycemic status associated with acromegaly.

Oxidative stress and TDH have become subjects of interest for many researchers. TDH parameters can be collectively measured more affordably and practically using the Erel and Neselioglu method [10]. Decreased serum thiol levels have been demonstrated in various diseases, including ankylosing spondylitis [39], Sjögren's syndrome [40], rheumatoid arthritis [41], Graves' disease [42], subacute thyroiditis [43], celiac disease [44], Behçet's disease [45], vitiligo patients [46], Guillain–Barre Syndrome [47], and myelodysplastic syndrome [48], compared to healthy controls. While inflammation and autoimmunity can increase OS, elevated OS can also trigger inflammation and autoimmunity [45].

Ischemia-modified albumin is a biochemical index that reflects increased OS in cells experiencing hypoxia and ischemia [49]. It is considered a diagnostic marker for coronary artery disease [50]. Increased serum IMA levels, compared to healthy controls, have been demonstrated in conditions such as diabetic ketoacidosis [51], atopic dermatitis [52], rheumatic diseases [53], congestive heart failure [54], and acute traumatic brain injury [55]. In our study, we also demonstrated increased serum IMA levels in patients with acromegaly, which may be associated with increased OS and a higher risk of cardiovascular system diseases in acromegaly.

The present study has some limitations, one of which is its cross-sectional design. Patients in remission and those with active disease could have been better matched; for example, the duration of the disease and treatments was not similar. The sample size of the study could have been larger; however, compared to similar studies, the current study has the largest sample size. A comparative evaluation of patients as treatment-naive at the time of diagnosis and post-treatment, whether surgical or medical, could have added more value to the study. Treatments for acromegaly and some individual differences are potential confounders, as active smokers were not included in the study, but the status of ex-smokers could be an influential factor, and possible differences in the dietary habits of the participants are also likely.

In conclusion, this study is the first in the literature to investigate serum TDH parameters and IMA levels in acromegalic patients. We observed a decrease in serum thiol levels, an indicator of the antioxidant system, and an increase in serum IMA levels, a marker of OS, suggesting the presence of elevated OS in the acromegalic process. The OS pathway may contribute to the development of acromegaly or to disease-related complications and comorbidities. Further research is needed to explore the various mechanisms and different pathways underlying the relationship between acromegaly and OS.

## Data Availability

No datasets were generated or analysed during the current study.
